# Supplementary material for: Centrosome Formation in the Bovine Early Embryo
Source: Cells. 2023 May 7;12(9):1335. doi: 10.3390/cells12091335 (PMC10177215; doi:10.3390/cells12091335)
Supplement: Supplementary file 1 [file cells-12-01335-s001.zip › cells-2361598-SM.pdf]

cells MDPI

Supplementary data

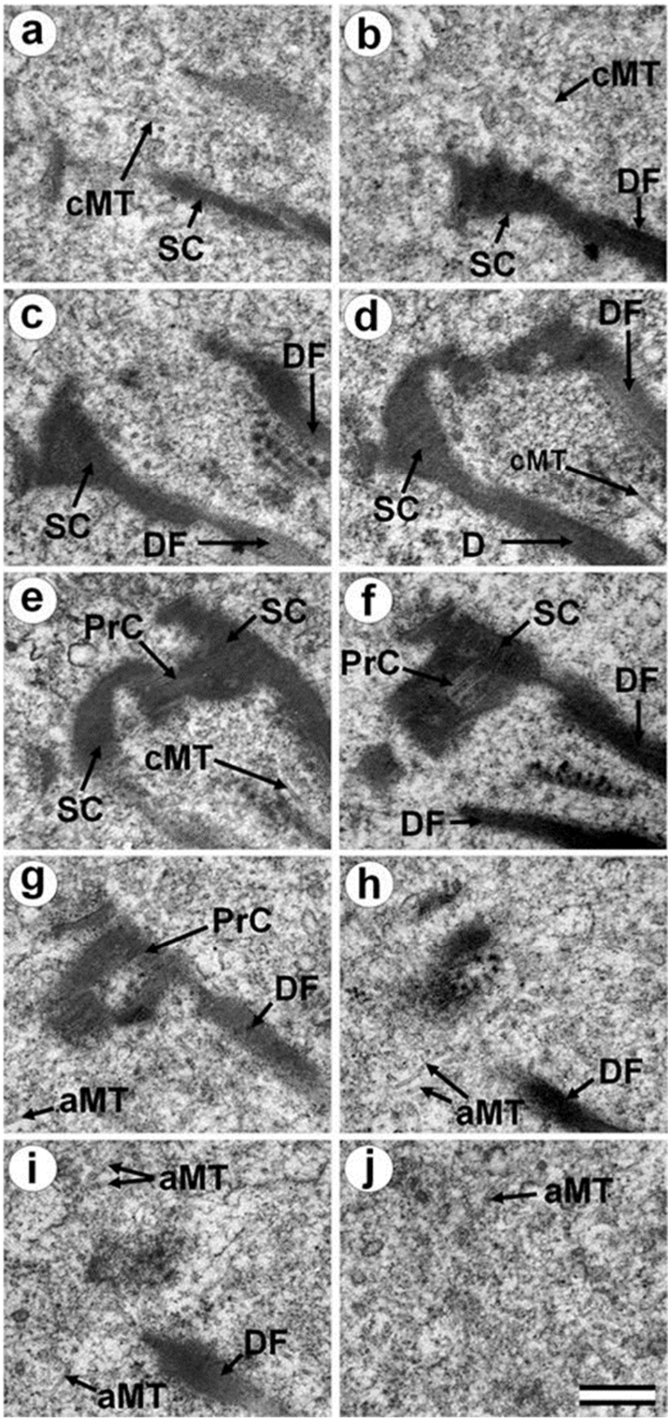

Figure S1. Ten serial sections of the neck portion of the sperm flagellum in the bovine zygote at the two pronuclei stage prior to convergence, 24 hours after fertilization. cMT – central MT of flagellum; aMT – MT of aster; DF – dense fibers; PrC – proximal centriole; SC – striated column. Scale bar: 300 nm.

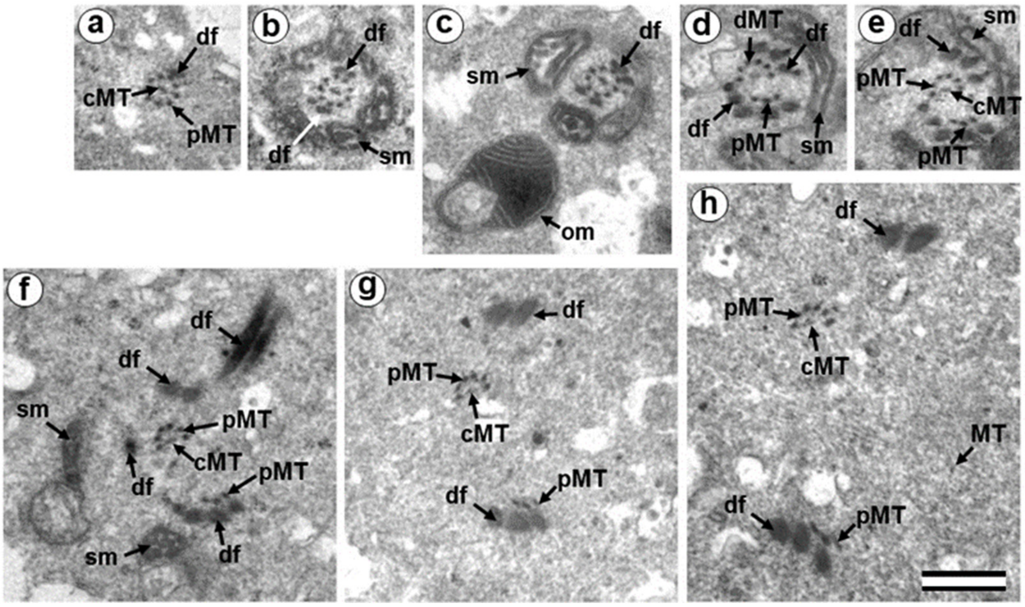

Figure S2. Serial sections of the sperm flagellum in the bovine zygote, 24 hours after fertilization. a – section № 395; b – section № 406; c – section № 423; d – section № 498; e – section № 501; f – section № 506; g – section № 512; h – section № 518; cMT – central MT of flagellum; df – dense fibers; MT – MT of cytoplasm; sm – mitochondria of the sperm origin; om – mitochondria of the oocyte origin; pMT – peripheral MT doublets of sperm flagellum;. Scale bar: 500 nm.

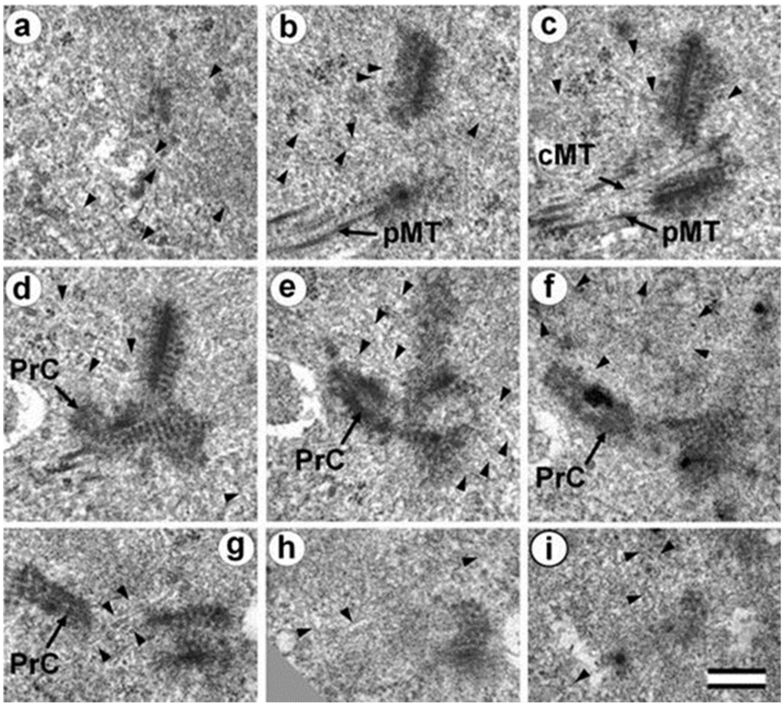

Figure S3. *Nine serial sections of the neck portion of the sperm flagellum in the bovine zygote at pronuclei convergence, 24 hours after fertilization. cMT – central MT of flagellum; PrC – proximal centriole; astral MTs are indicated by arrowheads. Scale bar: 300 nm.*

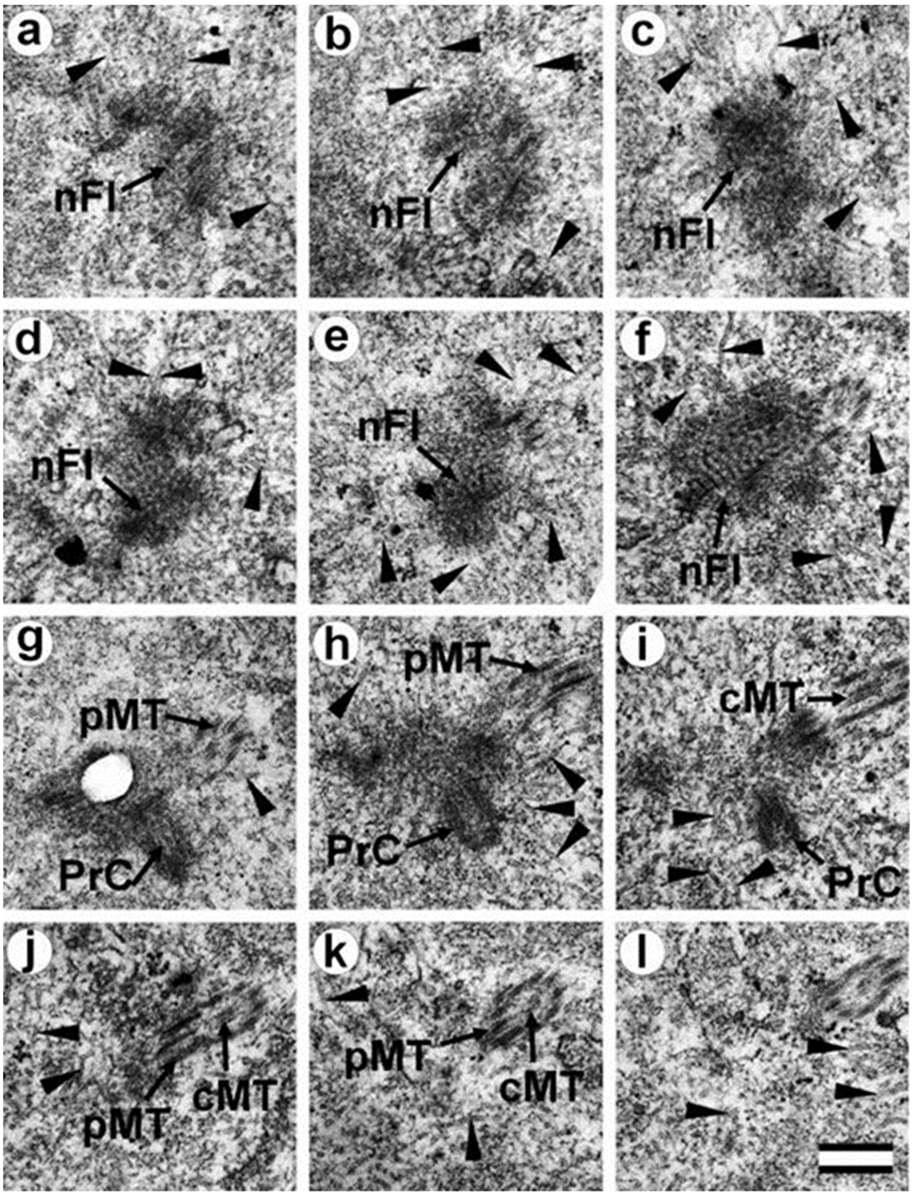

Figure S4. *Twelve serial sections of the neck portion of the sperm flagellum in the bovine embryo at prophase of the first cleavage, 24 hours after fertilization. nFl – neck portion of flagellum; PrC – proximal centriole; astral MTs are indicated by arrowheads (only part of MTs are shown). Scale bar: 300 nm. Note:* ***Fig. S4g*** *shows a defect in the Formvar film.*

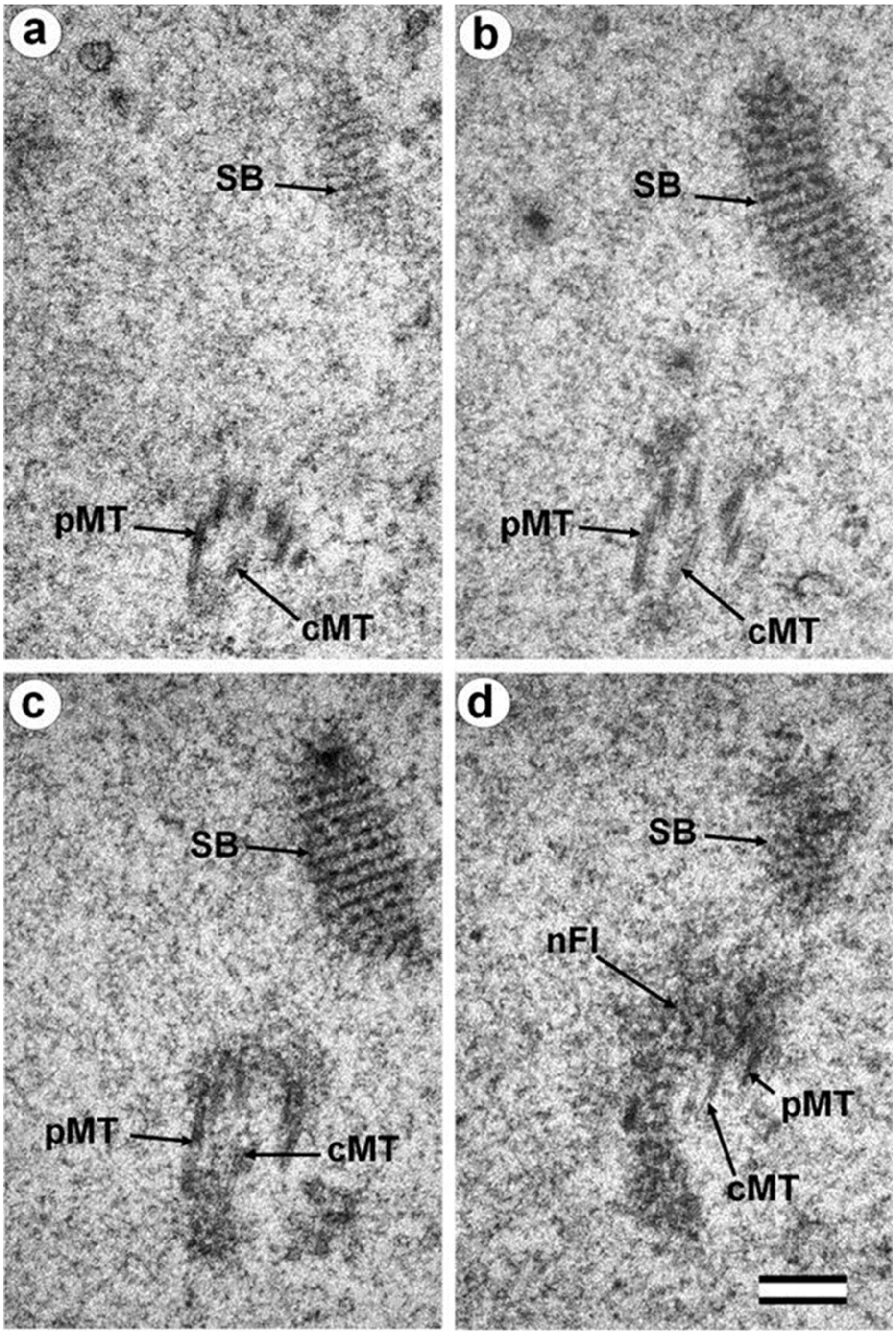

Figure S5. *(first part of series) Bovine embryo at the first cleavage (the late anaphase-early telophase), 30 hours after fertilization. Twenty serial sections of the pole associated with the neck portion of the sperm flagellum. cMT – central MT of sperm flagellum; nFl – neck portion of sperm flagellum; pMT – peripheral MT doublets of sperm flagellum; SB – "striated body". Scale bar: 200 nm.*

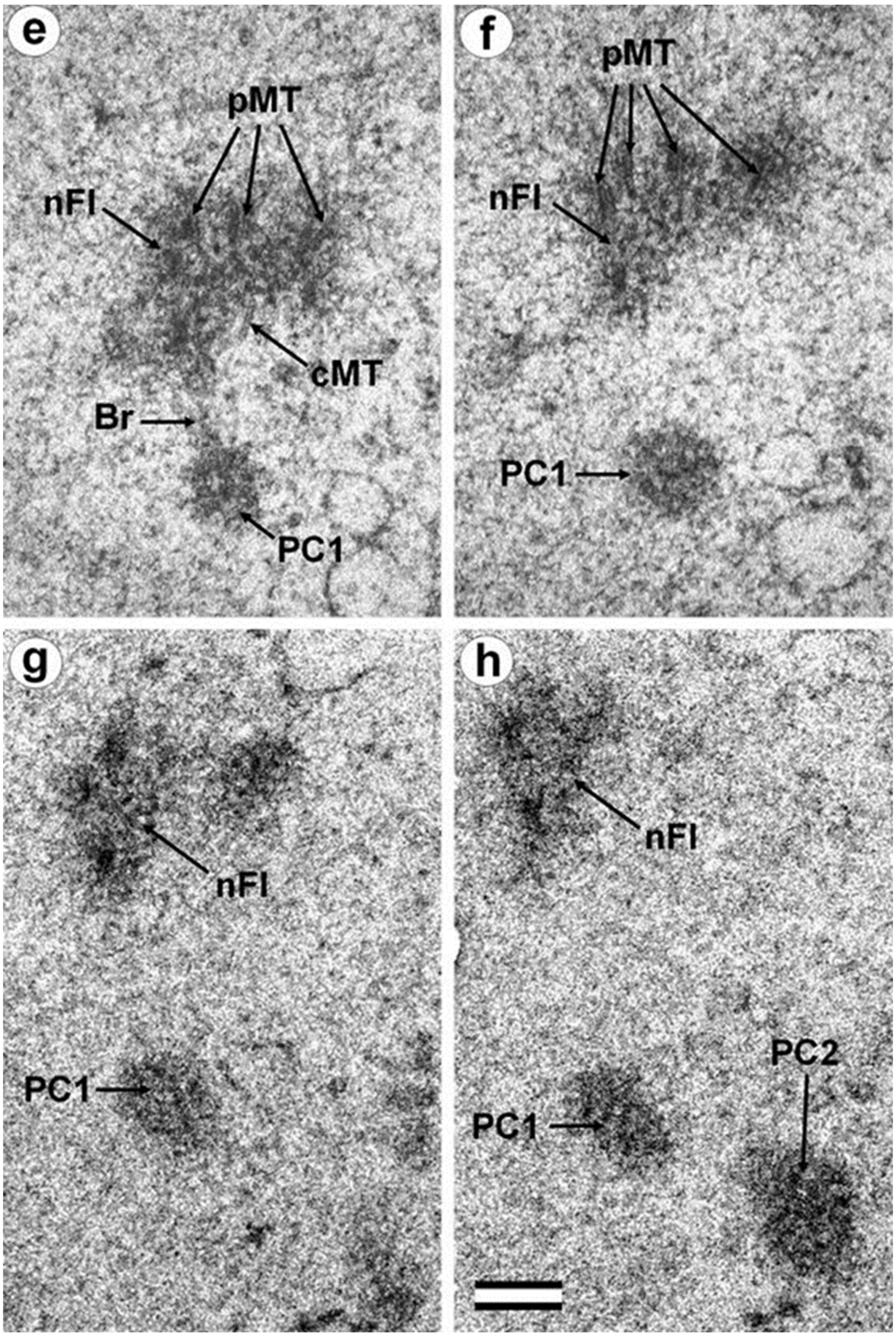

Figure S5. *(second part of series) Bovine embryo at the first cleavage (the late anaphase-early telophase), 30 hours after fertilization. Twenty serial sections of the pole associated with the neck portion of the sperm flagellum. Br – "bridge" between terminal portion of sperm flagellum and polar corpuscle 1; cMT – central MT of sperm flagellum; nFl – neck portion of sperm flagellum; PC1 – polar corpuscle 1; PC2 – polar corpuscle 2; sMT – MT of spindle. Scale bar: 200 nm.*

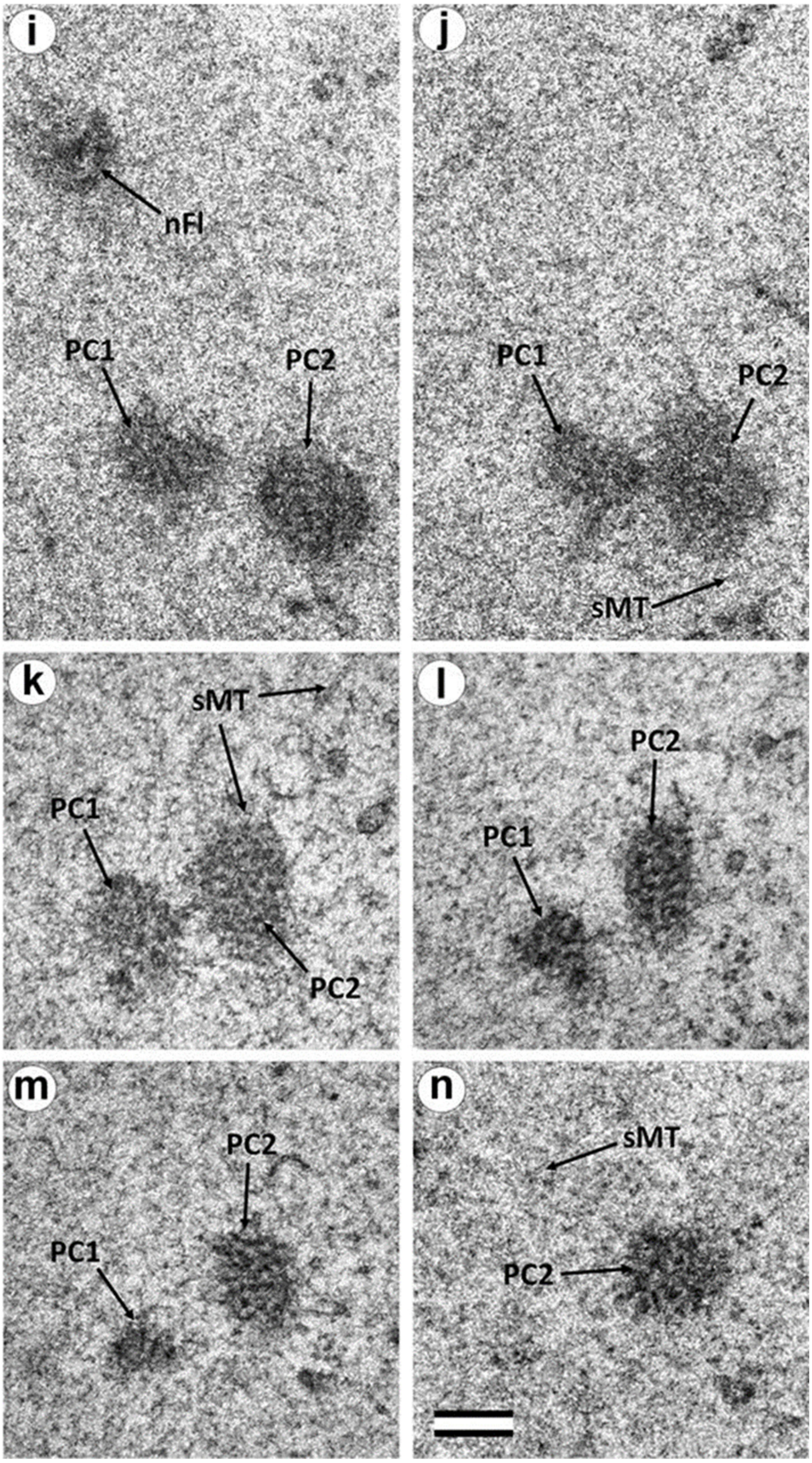

Figure S5. (third part of series) Bovine embryo at the first cleavage (the late anaphase-early telophase), 30 hours after fertilization. Twenty serial sections of the pole associated with the neck portion of the sperm flagellum. nFl – neck portion of sperm flagellum; PC1 – polar corpuscle 1; PC2 – polar corpuscle 2; sMT – MT of spindle. Scale bar: 200 nm.

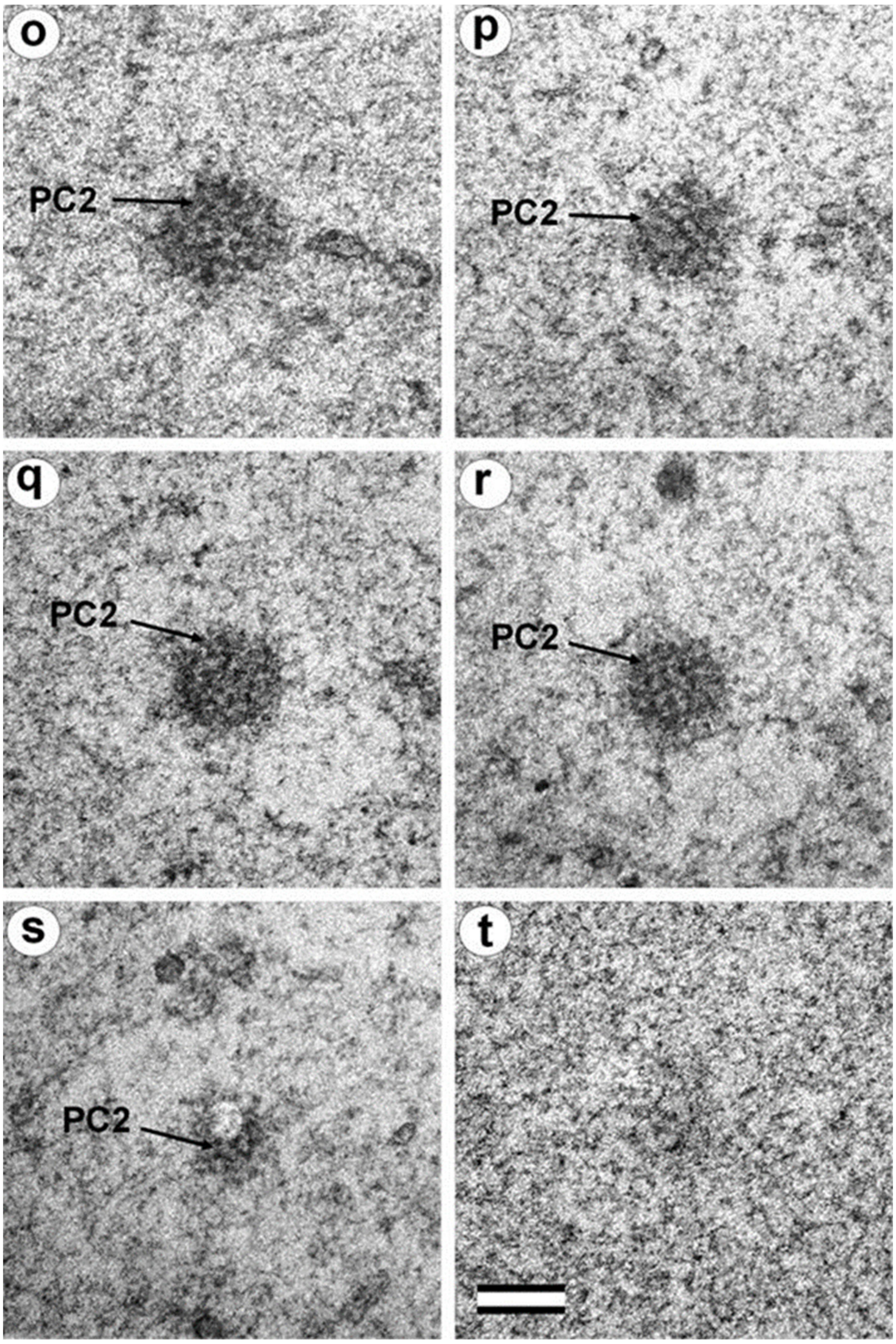

Figure S5. *(fourth part of series) Bovine embryo at the first cleavage (the late anaphase-early telophase), 30 hours after fertilization. Twenty serial sections of the pole associated with the neck portion of the sperm flagellum. PC2 – polar corpuscle 2. Scale bar: 200 nm.*

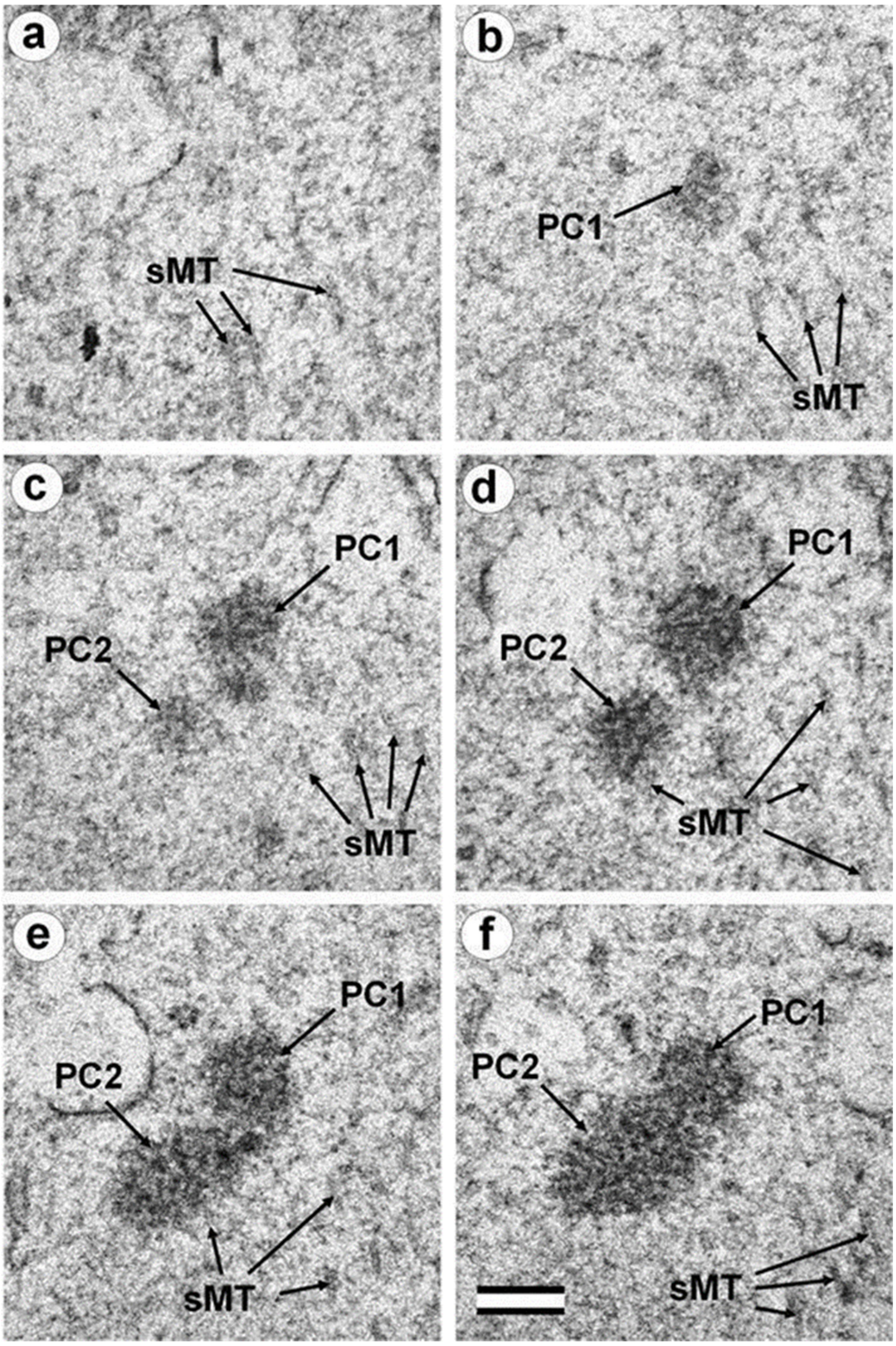

Figure S6. (first part of series) Bovine embryo at the first cleavage (the late anaphase-early telophase), 30 hours after fertilization. Ten consecutive sections of the pole opposite the pole connected to the neck of the sperm flagellum. PC1 – polar corpuscle 1; PC2 – polar corpuscle 2; sMT – MT of spindle. Scale bar: 200 nm.

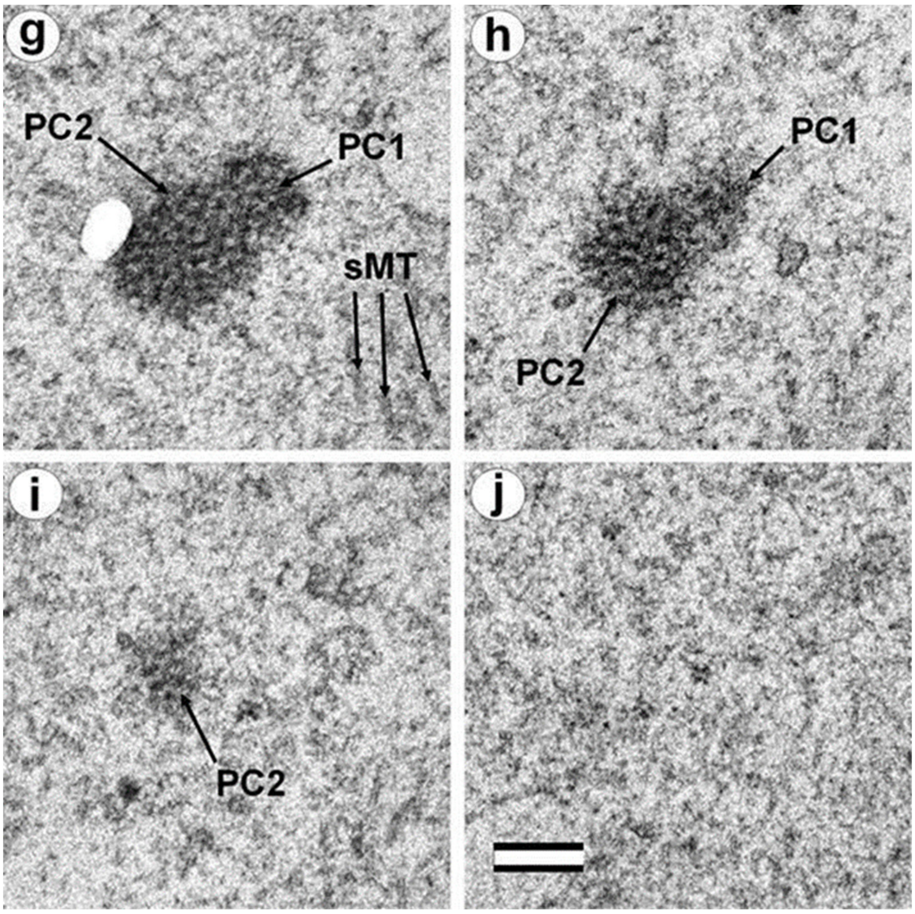

Figure S6. (second part of series) Bovine embryo at the first cleavage (the late anaphase-early telophase), 30 hours after fertilization. Ten consecutive sections of the pole opposite the pole connected to the neck of the sperm flagellum. PC1 – polar corpuscle 1; PC2 – polar corpuscle 2; sMT – MT of spindle. Scale bar: 200 nm.

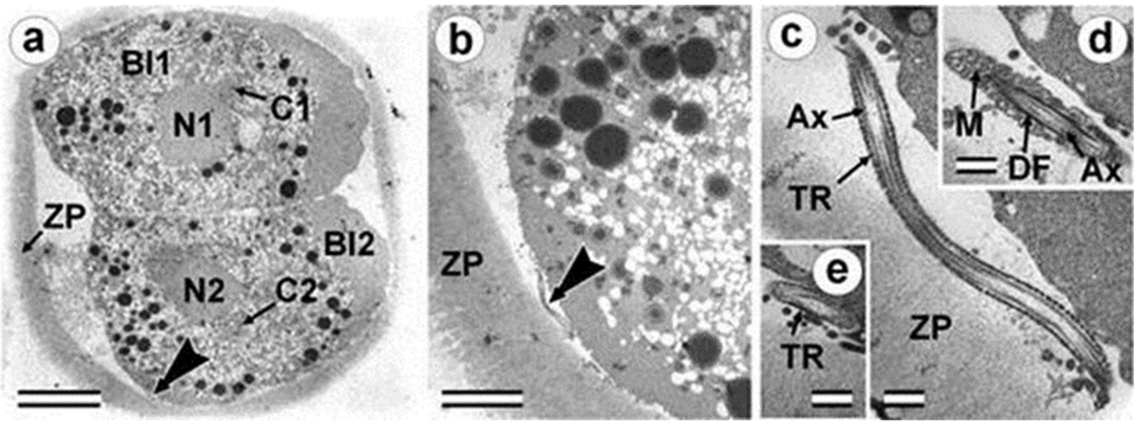

Figure S7. Two-cell bovine embryo I with sperm tail axoneme, 30 hours after fertilization. a) General view of the embryo at low magnification; b) flagellar area near blastomere Bl2; c–e) flagellar sections at different levels; c, e) principal piece of flagellum; d) middle piece of flagellum. The large arrow indicates the flagellar region. Bl1 – blastomere No. 1; Bl2 – blastomere No. 2; C1 – centrosomal region of blastomere Bl1; C2 – centrosomal region of blastomere Bl2; N1 – nucleus of blastomere Bl1; N2 – nucleus of blastomere Bl2; ZP – Zona pellucida; Ax – axoneme of flagellum; TR – transverse ribs of fibrous sheath in principal piece of flagellum; M – mitochondria of spermatozoon; DF – dense fibers of middle piece of flagellum. Scale bars: a - 20 µm; b - 1 µm; c–e - 0.3 µm.

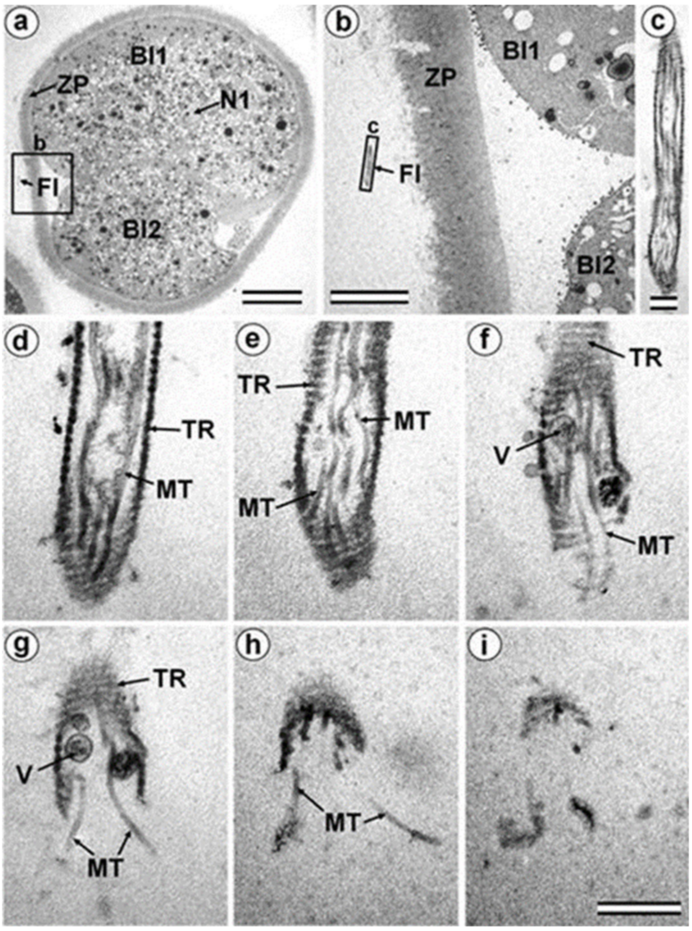

Fig. S8. *Two-cell embryo II with flagellum outside the zona pellucida, 30 hours after fertilization. a) General view of the embryo at low magnification; b) the flagellar region; c) flagellum at medium magnification; d–i) serial sections of the anterior broken end of the sperm flagellum. Bl1 – blastomere No. 1; Bl2 – blastomere No. 2; Fl – flagellum; MT – microtubules of axoneme; N1 – nucleus of blastomere No. 1; TR – transverse ribs of fibrous sheath in principal piece of sperm flagellum; V – membrane vesicle; ZP – Zona pellucida. Scale bars: a - 20 µm; b - 5 µm; c–i - 0.3 µm.*

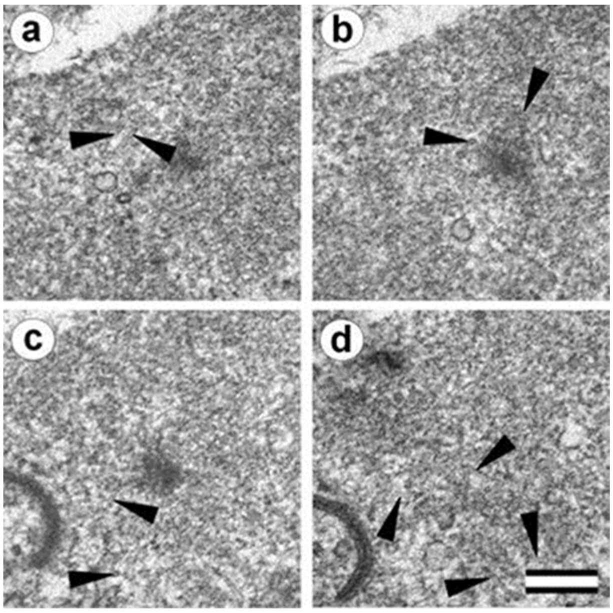

Figure S9. Three-cell bovine embryo at prophase of the second cleavage, 30 hours after fertilization. a, b) two consecutive sections before the section shown in Fig. 14c; c, d) two consecutive sections after the section shown in Fig. 14k. Arrowheads indicate MTs in cytoplasm. Scale bar: 0.2 μm.

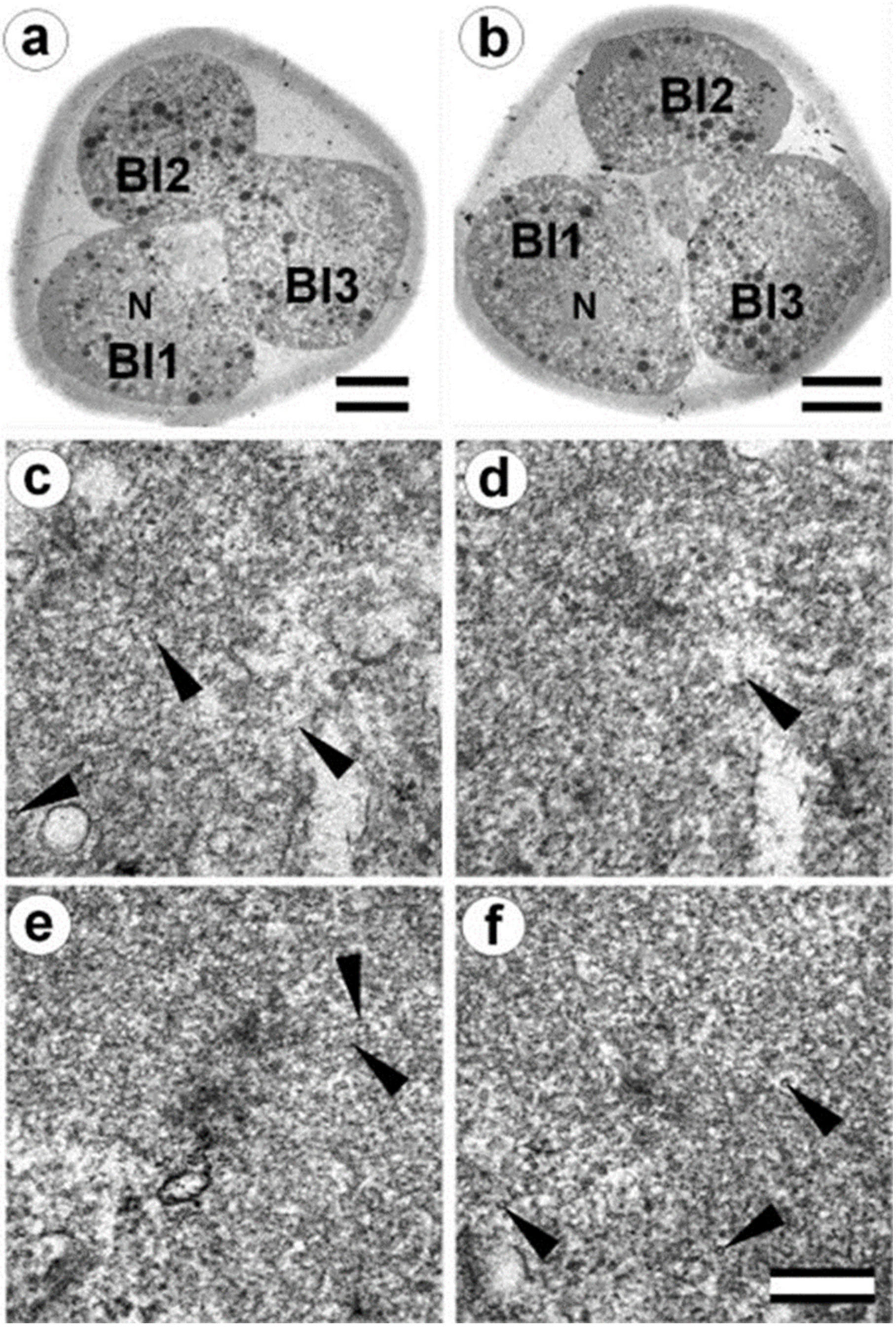

Figure S10. *Three-cell bovine embryo at cytotomy after the second cleavage, 30 hours after fertilization. a, b) general views at low magnification of embryonic sections at two different levels, showing the passage of cytotomy; c, d) two consecutive sections before the section shown in* ***Fig. 15c;*** *d, f) two consecutive sections after the section shown in* ***Fig. 15k.*** *Bl1 – blastomere No. 1; Bl2 – blastomere No. 2; Bl3 – blastomere No. 3; N – nucleus. Scale bars: a, b - 20 µm; c–f - 0.2 µm.*

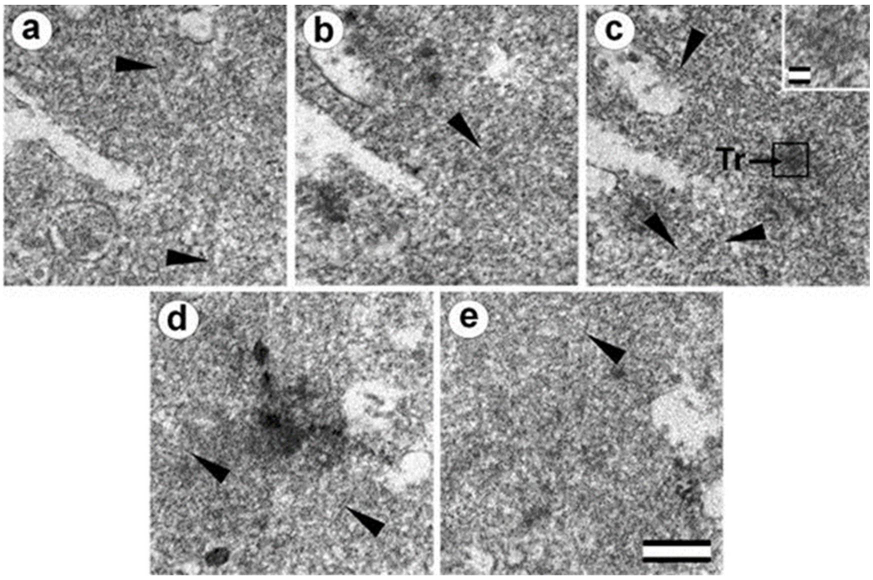

Figure S11. *Three-cell bovine embryo at cytotomy after the second cleavage, 30 hours after fertilization. a–c) three consecutive sections before the section shown in* ***Fig. 16c;*** *d, f) two consecutive sections after the section shown in* ***Fig. 16k.*** *Tr – separated triplets of MT; arrowheads indicate MTs in cytoplasm. Scale bar: 0.2 µm.*

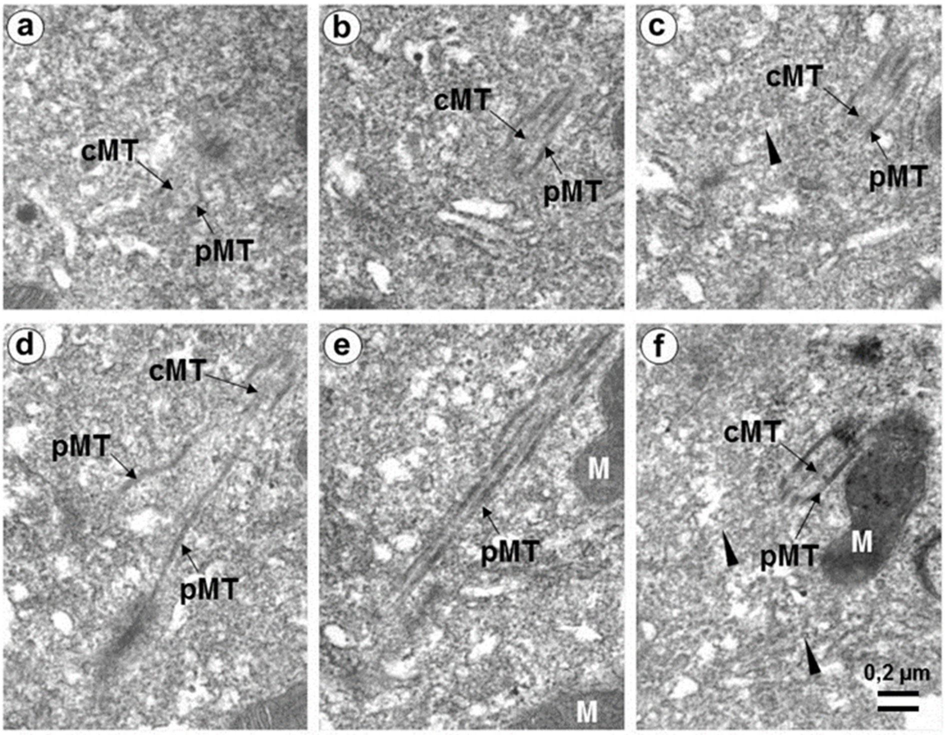

Figure S12. *The sperm flagellum axoneme in blastomeres of a 14-cell embryo with atypical centrosome and small procentriole, 36 hours after fertilization. a–f) six serial sections containing the sperm flagellum axoneme. cMT – central MT of axoneme; pMT – peripheral doublets of axoneme MT; M - mitochondria; arrowheads indicate MTs in cytoplasm. Scale bar: 0.2 µm. Sections after the section shown in* ***Figure 18l****.*
